# Prognostic Lnc-S100B-2 Affects Cell Apoptosis and Microenvironment of Colorectal Cancer through MLLT10 Signaling

**DOI:** 10.1155/2022/3565118

**Published:** 2022-01-25

**Authors:** Jianmei Yi, Feng Peng, Jingli Zhao, Xiaosong Gong

**Affiliations:** ^1^Department of General Surgery 2, Zhuzhou Central Hospital, Zhuzhou 412007, China; ^2^Operating Room, Zhuzhou Central Hospital, Zhuzhou 412007, China

## Abstract

Long noncoding RNA (LncRNA) is closely associated with the development of colorectal cancer (CRC). The chip data and clinical information of GSE104364 and GSE151021 were downloaded by GEOquery. Limma and Kaplan–Meier analysis were performed. Lnc-S100B-2 was obtained, and high expression of Lnc-S100B-2 was predicted to be associated with a lower survival rate. Online software was adopted to predict downstream regulatory genes, and miR-331-3p and Mixed Lineage Leukemia Translocated to 10 (MLLT10) were screened and verified. After silencing Lnc-S100B-2 and MLLT10, the proliferative activity of CRC cells decreased, and the apoptosis rate increased. At the gene and protein levels, the expressions of PCNA, Ki67, and Bcl-2 were decreased in the sh-Lnc-S100B-2 group, sh-MLLT10 group, and sh-Lnc-S100B-2 + sh-MLLT10 group, while the expressions of cleaved caspase 3, caspase 9, and Bax were increased. *In vivo*, the volume and mass of the tumor decreased in the sh-Lnc-S100B-2 + sh-MLLT10 group. Proliferation and apoptosis-related index (PCNA, Ki67, cleaved caspase 3, caspase 9, Bax, and Bcl-2) expression level was also altered. Meanwhile, the infiltration of immune cells (CD3 (-), CD16 (+), and CD11b (+) cells) decreased. The expressions of epithelial-mesenchymal transformation (EMT) related indicators (E-cadherin, N-cadherin, Vimentin, *β*-catenin, Snail, and Slug) were changed. E-cadherin and *β*-catenin were increased in the sh-Lnc-S100B-2 + sh-MLLT10 group, while N-cadherin, vimentin, snail, and slug were decreased. In conclusion, our study found that the expression of Lnc-S100B-2 was dysregulated in CRC. Lnc-S100B-2 could affect cell apoptosis and the microenvironment of CRC through regulating MLLT10.

## 1. Introduction

Colorectal cancer (CRC) is one of the most common malignant tumors in humans and the fourth deadliest cancer in the world, with nearly 900,000 deaths every year [1]. CRC has become a major global public health problem [2]. As previously described, CRC might develop in patients with distinct intestinal diseases such as inflammatory bowel diseases, microscopic colitis, and irritable bowel syndrome [3]. It might bring some difficulties to the diagnosis of CRC. Studies have shown that some progress has been made in diagnosing, treating, and preventing CRC. For example, colonoscopy's targeted screening and surveillance policy will curb the rising incidence of CRC [4]. Allium constituents are shown to modify the risk of colon cancer and reduce the mortality rates associated with this malignancy [5]. The poor prognosis of CRC patients remains a major problem [6]. CRC patients are usually diagnosed as advanced, with a poor prognosis and a low 5-year survival rate [7]. Previous studies have shown that the poor prognosis of CRC is related to molecular and gene changes [8]. Differential genes and molecules have essential research value in CRC [9,10].

Long noncoding RNAs (LncRNAs) are more than 200 nucleotides in length without protein-coding potential. LncRNAs are involved in regulating biological processes such as cell proliferation, differentiation, migration, and invasion [11–13] via mediating interactions between DNA and proteins, adsorbing microRNAs, and binding to proteins as decoys [14,15]. In recent years, studies on LncRNAs have attracted widespread attention. LncRNA interacts with cell metabolism (glucose metabolism, mitochondrial function, and oxidative stress) to affect cancer development [16]. In breast cancer and bladder cancer studies, LncRNAs can be used as prognostic markers for patients [17,18]. Similarly, in studies on CRC, prognostic LncRNAs have been found to promote or inhibit the growth, metastasis, invasion, and affect the microenvironment of CRC [9,19,20]. However, the role of Lnc-S100B-2 played in CRC cells, and the CRC microenvironment has never been reported previously.

Mixed Lineage Leukemia Translocated to 10 (MLLT10) is a transcriptional activator of gene expression. MLLT10 rearrangement is closely related to the development of leukemia. MLLT10 is one of the most common fusion partners of mixed-lineage leukemia (MLL, also known as KMT2A) in acute leukemia [21]. MLLT10 and IL3 are involved in gene rearrangement in patients with early T-cell precursor acute lymphoblastic leukemia [22]. Meanwhile, MLLT10 might be involved in the metastasis of non-small cell lung cancer [23]. The expression of MLLT10 is different in CRC [24]. However, the regulatory pathway of MLLT10 in CRC remains unclear.

In the study, we aimed to obtain prognostic LncRNA and their downstream regulatory genes through database screening and bioinformatics prediction. The expression of genes and their interrelationships were verified by experiments. Its functions were verified by *in vitro* and *in vivo* experiments. The study was expected to provide a biomarker and a promising therapeutic target for the treatment of CRC.

## 2. Methods

### 2.1. CRC Dataset and Bioinformatics Analysis

CRC datasets (GSE104364 and GSE151021) were downloaded from Gene Expression Omnibus (GEO) (http://www.ncbi.nlm.nih.gov/geo/). Among them, the GSE104364 dataset included CRC patients (*N* = 12) and normal controls (*N* = 6). The GSE151021 dataset included CRC patients (*N* = 4) and normal controls (*N* = 4). The original chip expression data and the corresponding clinical information were downloaded by GEOquery.

Limma was used to analyze LncRNA differentially expressed on chip data [25], selection criteria for | logFC | > 1 and *P* < 0.05. The R-package pheatmap was used to cluster the expression patterns of differentially expressed LncRNAs in the two groups, and a heatmap was drawn for visualization.

### 2.2. Clinical Specimens

CRC samples (*N* = 5) and matched adjacent tissues (*N* = 5) were randomly collected from Zhuzhou Central Hospital. Before participation, we obtained the informed consent of the study subjects.

### 2.3. Cell Culture and Transfection

Human CRC cell line HCT116 was purchased from Shanghai Zhong Qiao Xin Zhou Biotechnology Co., Ltd. Cells were cultured in DMEM medium containing 10% FBS with 1% penicillin-streptomycin solution (C0222, Beyotime, China) in an incubator at 37°C, 5% CO_2_, and saturated humidity.

The silenced plasmid (NC), Lnc-S100B-2 silenced plasmid (sh-Lnc-S100B-2), Lnc-S100B-2 overexpressed plasmid (oe-Lnc-S100B-2), and MLLT10 silenced plasmid (sh-MLLT10) were purchased from HonorGene. Briefly, 5 *μ*g plasmid was added to 250 *μ*L serum-free medium and mixed. Lipofectamine 2000 (Invitrogen, USA) was used for transfection according to the manufacturer's instructions. The specific groups were as follows: a control group (without any treatment), an NC group (NC was transfected), a sh-Lnc-S100B-2 group (sh-Lnc-S100B-2 was transfected), a sh-MLLT10 group (sh-MLLT10 was transfected), and a sh-Lnc-S100B-2+sh-MLLT10 group (sh-Lnc-S100B-2 and sh-MLLT10 were transfected).

### 2.4. RNA Isolation and Quantitative Real-Time PCR (qRT-PCR)

The Trizol method was used to isolate the total RNA from tissues and HCT116 cells. Briefly, 0.02 g tissues or 5 × 10^6^ cells were lysed with 1 mL Trizol. Isopropyl alcohol and ethanol were successively added for extraction and separation. 30 *μ*L sterile enzyme-free water was used to dissolve RNA precipitates. After detecting the RNA concentration, HiFiScript cDNA Synthesis Kit (CW2569 M, CWBIO, China) and miRNA cDNA Synthesis Kit (CW2141S, CWBIO, China) were used reverse transcription with a 20 *μ*L reverse transcription reaction system. SYBR-Green PCR Master Mix (CW2601S, CWBIO, China) was used for PCR amplification using the 30 *μ*L amplification system. 40 cycles were amplified. 2^−ΔΔCt^ was applied to calculate RNA expression levels. The sequences of primers used in the study were listed at Table 1. The expression of U6 and *β*-actin was applied as control.

### 2.5. Plate Clone Formation Assay

As previously described, the plate clone formation assay was adopted to detect cell proliferation [26]. Briefly, cells were digested with 0.25% trypsin (C0201, Beyotime) and cultured for 14 days. The cells were fixed with 4% paraformaldehyde ((N1012, NCM Biotech) for 15 min and stained with crystal violet (G1062, Solarbio) for 30 min. A microplate reader (MB-530, HEALES) was adopted to measure the cell colony number, and pictures were taken.

### 2.6. Western Blot

The RIPA buffer (P0013 B, Beyotime) was used to extract proteins by lysing cells and tissues. The SDS-PAGE gel was used to separate the proteins. The proteins were transferred to the nitrocellulose membrane. 5% skimmed milk was used to block the membrane at 4°C overnight. The membranes were incubated with primary antibodies or secondary antibodies at room temperature (RT) for 90 min. The antibodies used were as follows: anti-*β*-actin (1 : 5000, 66009-1-Ig, proteintech), anti-PCNA (1 : 2000, 10205-1-AP, proteintech), anti-Ki67 (1 : 1000, 27309-1-AP, proteintech), anti-cleaved caspase 3 (1 : 1000, 9664S, CST), anti-caspase 9 (1 : 500, bs-20773R, Bioss Antibodies), anti-Bax (1 : 1000, ab32503, abcam), anti-Bcl-2 (1 : 1000, 12789-1-AP, proteintech), anti-E-cadherin (1 : 1000, 20874-1-AP, proteintech), anti-*β*-catenin (1 : 1000, bs-1165R, Bioss Antibodies), anti- N-cadherin (1 : 2000, 22018-1-AP, proteintech), anti-vimentin (1 : 2000, 10366-1-AP, proteintech), anti-Snail (1 : 1000, 13099-1-AP, proteintech), anti-Slug (1 : 1000, #9585, CST), HRP goat anti-mouse IgG (1 : 5000, SA00001-1, proteintech), and HRP goat anti-rabbit IgG (1 : 6000, SA00001-2, proteintech). Proteins were detected by Western Bright ECL kit (K-12045-D50, advansta). The expression of *β*-actin was applied as control.

### 2.7. Flow Cytometry

Apoptosis analysis was as follows. The cells were digested by trypsin without EDTA. Cells were washed twice by PBS and centrifuged at 2000 rpm for 5 min. 500 *μ*L binding buffer was added to resuspend cells. After being mixed with 5 *μ*L Annexin V-FITC, 5 *μ*L propidium iodide (PI) was added to the cells and mixed and incubated for 10 min in the dark at RT. Flow cytometry (A00-1-1102, Beckman Coulter, USA) was used for observation and analysis.

Cell-cycle analysis was as follows. The cells were digested by trypsin and centrifuged at 800 rpm for 5 min. After being resuspended with 400 *μ*L PBS, 1.2 mL of 100% precooled ethanol was added, and the cells were placed at 4°C overnight. Cells were washed twice with 1 mL precooled PBS. Then, cells were fixed with 150 *μ*L PI staining solution and incubated for 30 min in the dark at 4°C. Flow cytometry (A00-1-1102, Beckman Coulter, USA) was applied to analyze the cell cycle.

Identification of CD3 (-) CD16 (+) cells and CD11b (+) cells was as follows. 1 × 10^6^ cells were resuspended with 200 *μ*L PBS volume. Cells were incubated with 5 *μ*L CD3 (12-0038-42, eBioscience), CD16 (17-0168-42, eBioscience), or CD11b (12-0118-42, eBioscience) for 30 min in the dark. Cells were washed twice by 1 mL PBS. 200 *μ*L PBS was added to resuspend cells. After filtration with a nylon net, flow cytometry was used to detect the percent of CD3 (-) CD16 (+) cells and CD11b (+) cells.

### 2.8. Dual-Luciferase Reporter Assay

The online software miRDB (http://mirdb.org/index.html) was used to predict the target gene of miR-331-3p. Dual-luciferase reporter assay was used to identify the correlation between miR-331-3p and its target gene MLLT10. Briefly, 293A cells, MLLT10-wt plasmids, and MLLT10-Mut plasmids were purchased from HonorGene. MiR-331-3p mimics and mimic NC were purchased from Shanghai GenePharma Co., Ltd. The MLLT10-wt or MLLT10-Mut or miR-331-3p mimics or mimic NC were cotransfected into precultured 293A cells using Lipofectamine 2000. After 48 h, the luciferase activity was analyzed with the Dual Luciferase Reporter Assay System (Promega, USA).

### 2.9. Animal Experiments

Male BALB/*c* nude mice (*N* = 24) were purchased from Human SJA Laboratory Animal Co., Ltd. As previously mentioned [27], animal models were constructed. Briefly, mice were fed adaptively for a week with normal food, water, and light. Stable HCT116 cells were cultured after NC, sh-MLLT10, and sh-Lnc-S100B-2 transfection. After the cells had grown to about 80% fusion, they were digested with trypsin and counted. 200 *µ*L PBS containing 2 × 10^6^ HCT116 cells was injected into the right lower flank of 6–8 weeks old mice. They were randomly divided into four groups: the NC group, the sh-MLLT10 group, the sh-Lnc-S100B-2 group, and the sh-Lnc-S100B-2 + sh-MLLT10 group, with 6 rats in each group. After 35 days of normal feeding, the mice were euthanized humanely. The tumor body was taken, and the tumor volume was measured (volume = (widths × width × length)/2).

### 2.10. Immunohistochemistry (IHC)

Briefly, after 12 hours of baking at 60°C, the paraffin slices were dewaxed. After heating for antigenic repair, 1% periodic acid was used to inactivate endogenous enzyme activity. After incubation with anti-caspase 3 (1 : 200, 19677-1-AP, proteintech) at 4°C overnight, 100 *µ*L anti-rabbit IgG was inoculated at 37*°*C for 30 min. After DAB color development, the hematoxylin was counterstained for 10 min. Then, the sections were sealed with the neutral resin and observed with a light microscope.

### 2.11. Immunofluorescence (IF) Assay

The expression of CD3, E-cadherin, and vimentin in tissues was determined by IF. Briefly, after heating for antigenic repair, the sample was treated with hydrogen boride solution and Sudan black dye. 10% serum and 5% BSA were used to seal the sample for 60 min. Anti-CD3 (1 : 50, 17617-1-AP, proteintech), anti-E-cadherin (1 : 50, 20874-1-AP, proteintech), and anti-vimentin (1 : 50, 10366-1-AP, proteintech) were incubated overnight at 4°C, and anti-rabbit -IgG labeled fluorescent antibodies were incubated at 37°C for 90 min. Nuclear DNA was labeled with DAPI (blue). Cells were analyzed with a fluorescence microscope.

### 2.12. Statistics Analysis

Data were analyzed using the GraphPad Prism 8.0.1 and presented as the mean ± SD. Kaplan–Meier analysis and log-rank test were adopted to analyze the survival time of patients. Correlation between the expression of miR-331-3p and Lnc-S100B-2 was analyzed by Pearson's correlation analysis. Paired *t*-test, one-way ANOVA or two-way ANOVA with Tukey's multiple comparisons test were performed to evaluate the statistical significance. *P* < 0.05 was considered to indicate a statistically significant difference.

## 3. Results

### 3.1. Lnc-S100B-2 Is Highly Expressed with a Poor Prognosis in CRC

**Figure 1:**
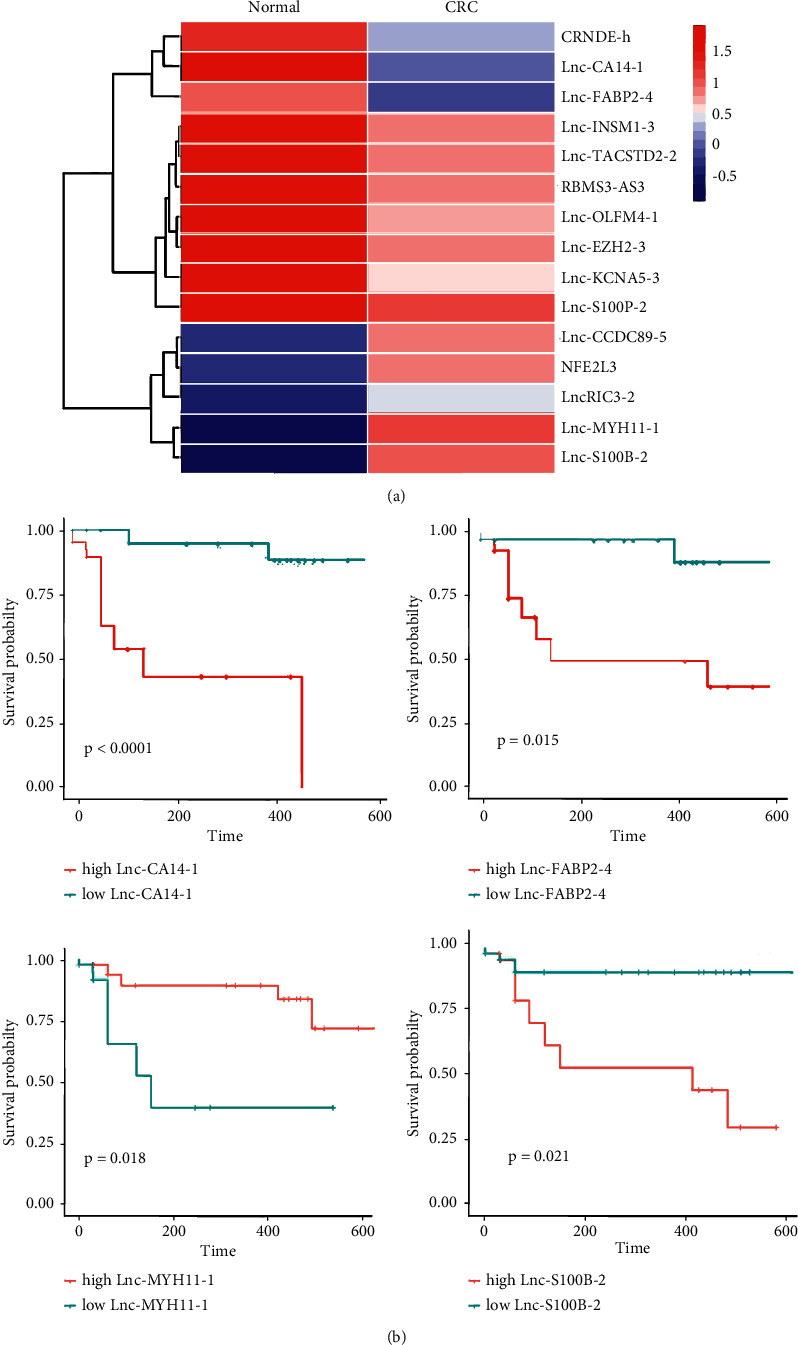
Expressions of differential LncRNA and prognosis in CRC. (a) The expression of differential LncRNAs in GEO. Blue turns red, indicating increased gene abundance. (b) Survival prediction analysis of the Lnc-CA14-1, Lnc-FABP2-4, Lnc-MYH11-1, and Lnc-S100B-2 in high and low groups.

To obtain differential LncRNAs in CRC, we analyzed the expression profiles of LncRNAs in the GSE104364 and GSE151021 datasets. We found a series of differentially expressed LncRNAs in CRC (Figure 1(a). Kaplan–Meier analysis showed that the survival curve of Lnc-CA14-1, Lnc-FABP2-4, Lnc-MYH11-1, and Lnc-S100B-2 was *P* < 0.05 (Figure 1(b)). Higher Lnc-S100B-2 level was associated with poorer survival. These results suggested that Lnc-S100B-2 might be involved in the prognosis of CRC.

### 3.2. Lnc-S100B-2 Affects the Proliferation and Apoptosis of HCT116 Cells

**Figure 2:**
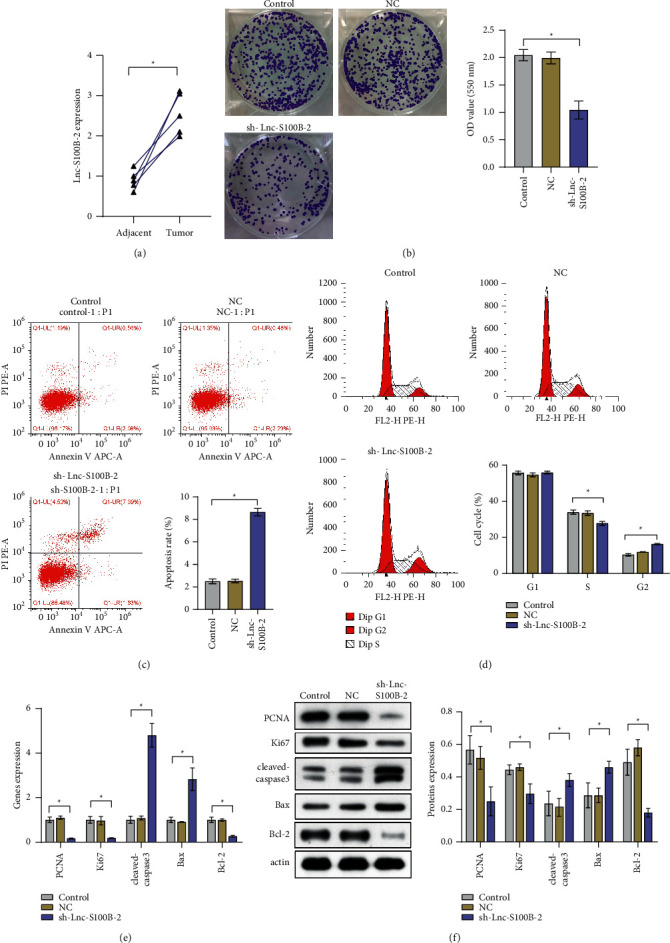
Effects of Lnc-S100B-2 in CRC cells. (a) Expression of Lnc-S100B-2 between adjacent and tumor tissues. (b) The proliferation of HCT116 cells by plate clone formation assay. (c) The apoptosis rate of HCT116 cells. (d) Cell cycle analysis of HCT116 cells. (e), (f) Expressions of PCNA, Ki67, cleaved caspase 3, Bax, and Bcl-2 at the gene and protein levels. ^∗^*P* < 0.05, paired *t*-test and one-way ANOVA.

We randomly collected 5 pairs of tumor and matched adjacent tissues. Clinical samples were used to verify the level of Lnc-S100B-2. The paired *t*-test (Figure 2(a)) were consistent with those of predicting results (Figure 1(a)). The expression of Lnc-S100B-2 was significantly upregulated in tumor tissues. The results of plate clone formation assay showed that the activity of HCT116 cells was decreased when Lnc-S100B-2 was inhibited (Figure 2(b)). Apoptosis results proved that Lnc-S100B-2 was positively correlated with CRC cell activity (Figure 2(c)). Knockdown of Lnc-S100B-2 resulted in cell stagnation in the G2 phase (Figure 2(d)). Expression of proliferation (PCNA and Ki67) and apoptosis-related indicators (cleaved caspase 3, Bax, and Bcl-2) at the gene and protein levels was identified (Figure 2(e) and 2(f)). Expressions of PCNA, Ki67, and Bcl-2 decreased in the sh-Lnc-S100B-2 group compared to the control group, while cleaved caspase 3 and Bax were the opposite. Combined with the above results, the expression of Lnc-S100B-2 in CRC might affect cell proliferation and apoptosis.

### 3.3. Lnc-S100B-2 Regulates MLLT10 in CRC

**Figure 3:**
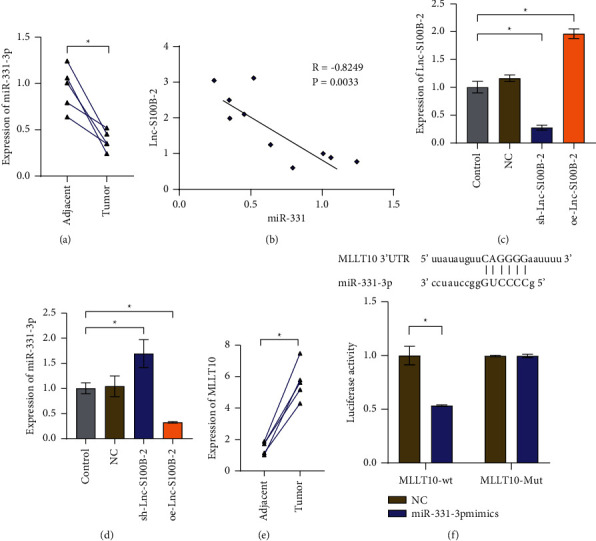
Lnc-S100B-2 regulates the expression of MLLT10 by miR-331-3p. (a) Expression of miR-331-3p between adjacent and tumor tissues. (b) Pearson's correlation analyzed the correlation between miR-331-3p and Lnc-S100B-2. (c), (d) Expression of Lnc-S100B-2 and miR-331-3p. (e) Expression of MLLT)10 between adjacent and tumor tissues. (f) Dual-luciferase reporter analysis of miR-331-3p and MLLT10. ^∗^*P* < 0.05, paired *t*-test and one-way ANOVA.

Next, we validated the expression of the downstream gene of Lnc-S100B-2. MiR-331-3p was decreased in cancer tissue (Figure 3(a)). Pearson's analysis showed that the levels of miR-331-3p were significantly negatively correlated with Lnc-S100B-2 (Figure 3(b)). The expression of miR-331-3p increased or decreased with the decrease or increase of Lnc-S100B-2 (Figures 3(c) and 3(d)). These results hinted that Lnc-S100B-2 could regulate the levels of miR-331-3p. Meanwhile, the expression of MLLT10 was higher in tumors than in adjacent mucosa (Figure 3(e)). The online software miRDB (http://mirdb.org/index.html) was used to predict the target gene of miR-331-3p. Dual-luciferase reporter assay results showed that miR-331-3p targeted MLLT10 (Figure 3(f)). The above experimental results suggested that Lnc-S100B-2 might regulate the expression of MLLT10 through miR-331-3p.

### 3.4. MLLT10 Could Promote HCT116 Cell Apoptosis

**Figure 4:**
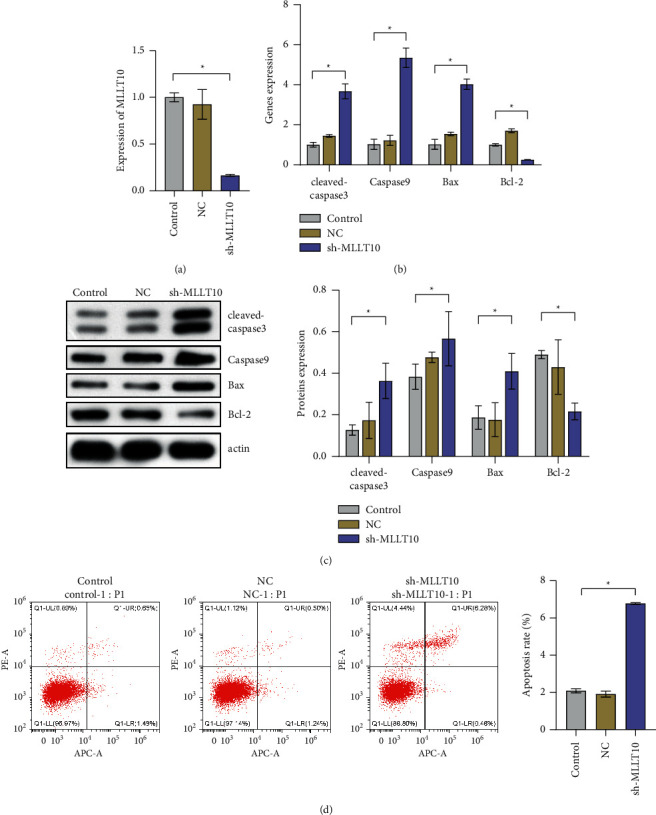
The expression of MLLT10 could affect cell apoptosis in CRC. (a) Expression of MLLT10. (b), (c) The expressions of cleaved caspase 3, caspase 9, Bax, and Bcl-2 at the gene and protein levels. (d) The apoptosis rate of HCT116 cells. ^∗^*P* < 0.05, one-way ANOVA.

To verify the role of MLLT10 in CRC, we stably transfected sh-MLLT10 in HCT116 cells. qRT-PCR results showed that sh-MLLT10 had good efficacy (Figure 4(a)). The expression of apoptosis-related indexes (cleaved caspase 3, caspase 9, Bax, and Bcl-2) significantly changed in the sh-MLLT10 group (Figure 4(b) and 4(c)). Meanwhile, the apoptosis rate of HCT116 cells also indicated that the expression of MLLT10 was negatively correlated with the apoptosis rate (Figure 4(d)). These results suggested that the levels of MLLT10 in CRC could affect cell apoptosis.

### 3.5. Effects of Lnc-S100B-2 and MLLT10 on the Development of CRC

**Figure 5:**
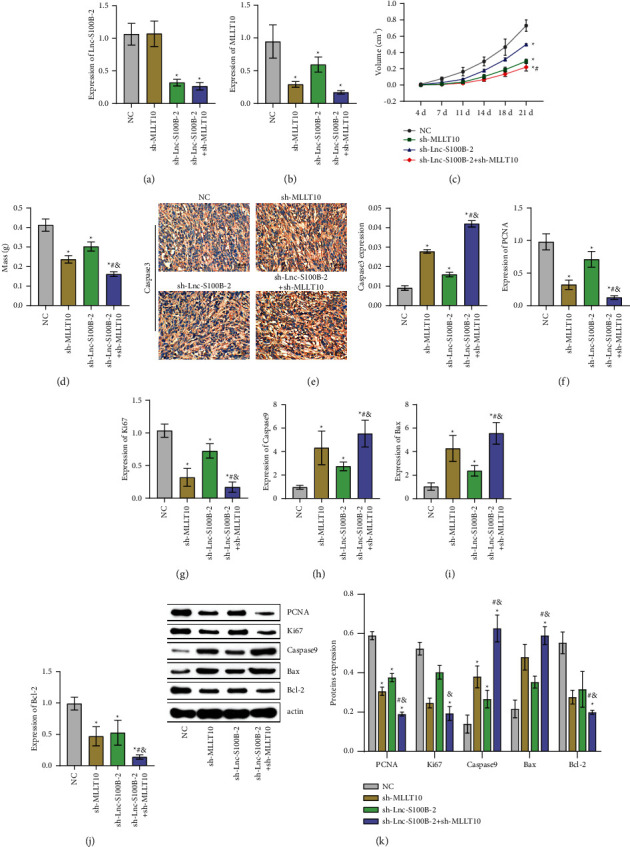
Effects of Lnc-S100B-2 and MLLT10 *in vivo*. (a, b) Expression of Lnc-S100B-2 and MLLT10. (c, d) Tumor volume and mass. (e) Expression of caspase 3 by IHC. (f–k) The expression of PCNA, Ki67, caspase 9, Bax, and Bcl-2 at the gene and protein levels. ^∗^*P* < 0.05 versus NC group, ^#^*P* < 0.05 versus sh-MLLT10 group, and ^&^*P* < 0.05 versus sh-Lnc-S100B-2 group, one-way ANOVA and two-way ANOVA.

Then, HCT116 cells transfected with NC, sh-MLLT10, sh-Lnc-S100B-2, or sh-Lnc-S100B-2+ sh-MLLT10 were subcutaneously injected into nude mice. The expression of Lnc-S100B-2 and MLLT10 was altered in the tumor (Figures 5(a) and 5(b)). Tumor volume and mass were significantly reduced after inhibition of Lnc-S100B-2 and MLLT10 (Figures 5(c) and 5(d)). After IHC staining of the tumor (Figure 5(e)), the expression of caspase 3 was significantly increased after sh-Lnc-S100B-2 and sh-MLLT10 treatment. Meanwhile, the sh-Lnc-S100B-2 + sh-MLLT10 group was markedly higher than the sh-MLLT10 group. These results suggested that Lnc-S100B-2 might regulate the expression of MLLT10 to affect cell apoptosis. At the gene and protein levels, the levels of proliferation (PCNA and Ki67) and apoptosis-related indexes (cleaved caspase 3, Bax, and Bcl-2) further suggested that Lnc-S100B-2 could affect the development of CRC by regulating the expression of MLLT10 (Figures 5(f)–5(k)). These results indicated that Lnc-S100B-2 might affect the proliferation and apoptosis of CRC cells by regulating MLLT10.

### 3.6. Effects of Lnc-S100B-2 and MLLT10 on Immune Cell Invasion and EMT in CRC

**Figure 6:**
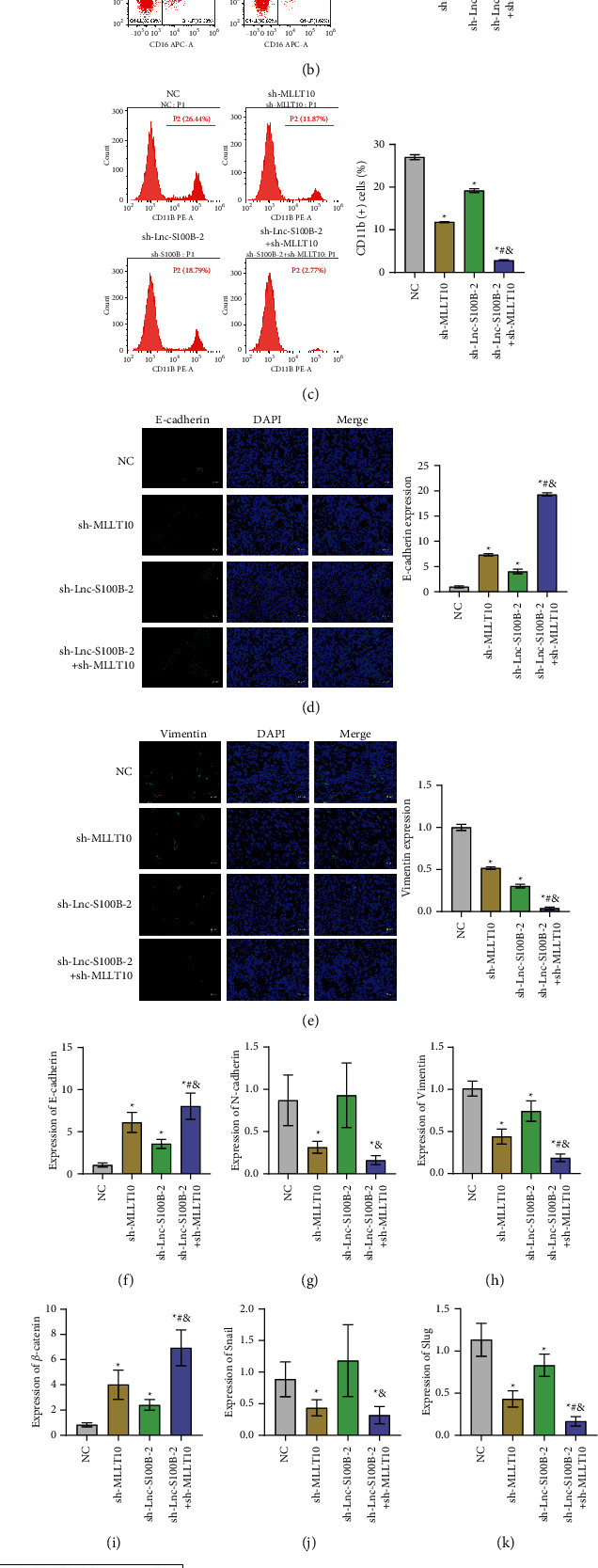
Effects of Lnc-S100B-2 and MLLT10 on the tumor microenvironment. (a) The expressions of CD3 were detected by IF assay. (b) The percent of CD3 (-) CD16 (+) cells was detected by flow cytometry. (c) The percent of CD11b (+) cells was analyzed by flow cytometry. (d, e) The expression E-cadherin and vimentin was analyzed by IF assay. (f–l) The expressions of E-cadherin, N-cadherin, vimentin, *β*-catenin, snail, and slug were detected by qRT-PCR and Western blot. ^∗^*P* < 0.05 versus NC group, ^#^*P* < 0.05 versus sh-MLLT10 group, ^&^*P* < 0.05 versus sh-Lnc-S100B-2 group, and one-way ANOVA.

Immune cell invasion and EMT are two essential components of the tumor microenvironment. To further explore the role of Lnc-S100B-2 and MLLT10 in CRC, we investigated the immune cell invasion and the degree of EMT in the tumor. CD3 expression was significantly decreased after sh-Lnc-S100B-2 and sh-MLLT10 treatment (Figure 6(a)). It suggested that the infiltration degree of lymphocytes in the tumor tissue was reduced. The number of CD3 (-) CD16 (+) cells and CD11b (+) cells were also significantly decreased with the silencing of Lnc-S100B-2 and MLLT10 (Figures 6(b) and 6(c)). These results suggested that regulation of Lnc-S100B-2 and MLLT10 might affect the abundance of immune cells in tumor tissues. In addition, the expression of E-cadherin was significantly increased in the sh-MLLT10 group compared with the other three groups. Vimentin is the opposite (Figure 6(e)). We examined the expression levels of EMT-related indicators (E-cadherin, N-cadherin, vvimentin, *β*-catenin, snail, and slug) at the gene and protein levels. The results showed (Figures 6(f)–6(l)) that E-cadherin and *β*-catenin were significantly increased in the sh-Lnc-S100B-2+sh-MLLT10 group, compared with the sh-Lnc-S100B-2 group and the sh-MLLT10 group, while N-cadherin, vimentin, snail, and slug were decreased considerably. It is suggested that Lnc-S100B-2 might affect the EMT of tumor cells through MLLT10, at least partially. Combined with the above experimental results, we found that the regulation of Lnc-S100B-2 and MLLT10 could affect the immune cell invasion and EMT in the tumor.

## 4. Discussion

In our study, Lnc-S100B-2 has obtained through Limma and Kaplan–Meier analysis in the CRC datasets (GSE104364 and GSE151021). At the cellular and animal levels, the effects of Lnc-S100B-2 and its downstream MLLT10 signaling on CRC have been identified.

Lnc-S100B-2 is a long noncoding RNA. Our study found that Lnc-S100B-2 was overexpressed in CRC. The expression of Lnc-S100B-2 could affect the proliferation, apoptosis, and EMT of CRC cells. The prognosis of CRC is closely related to EMT. Kaplan–Meier analysis showed that the overexpression of Lnc-S100B-2 predicted a poor prognosis in CRC. EMT is closely associated with poor prognosis of cancer patients, including gastric cancer [28], glioma [29], and bile duct cancer [30]. In bladder cancer, Cao *R.* et al. found that EMT, as a negative independent prognostic factor, had a tumor-promoting effect due to its related genetic characteristics [31]. These findings suggest that EMT in CRC may affect patient prognosis. At the same time, this verified our results from the side that Lnc-S100B-2 affected the prognosis of CRC through EMT of CRC cells.

Our study found that Lnc-S100B-2 might regulate the expression of MLLT10 through miR-331-3p. miRNA is also involved in CRC development and prognosis [32]. Lin et al. showed that miR-195-5p/NOTCH2 signaling could affect the polarization of M2-like tumor-associated macrophages by mediating tumor cell EMT [33]. Zhang Y et al. found that miR-17-5P could activate cancer-associated fibroblasts by regulating RUNX3/MYC/TGF-*β*1 signaling, influencing tumor microenvironment and promoting CRC development [34]. These results suggest that miRNA might influence the tumor microenvironment and CRC development by regulating the expression of downstream target genes.

Studies have shown that MLLT10 is often observed in acute myeloid and lymphoid leukemia, affecting its treatment and prognosis [35,36]. Previous studies have shown that inhibition of MLLT10 expression can affect the proliferation, migration, and invasion of non-small cell lung cancer cells [23]. It is similar to our findings. MLLT10 could affect the apoptosis level of CRC cells. The expression of apoptosis-related indicators (cleaved caspase 3, caspase 9, Bax, and Bcl-2) was altered with the silence of MLLT10. MLLT10 also has a particular regulatory effect on cell EMT and immune cell infiltration. After inhibiting the expression of MLLT10, the expression levels of EMT-related indicators (E-cadherin, N-cadherin, vimentin, *β*-catenin, snail, and Slug) changed. EMT is involved in the migration, invasion, and metastasis of cancer cells [37]. EMT is closely related to cell apoptosis. A negative correlation between apoptosis and EMT has been reported in ovarian cancer [38]. Vimentin can affect the apoptosis of SMMC-7721 cells in liver cancer studies [39]. Regulation of Snail1 expression can restore EMT and prevent ethanol-induced apoptosis of neural crest cells [40]. All these proved from the side that MLLT10 affects CRC cell apoptosis and EMT, with sure accuracy. Jing et al.'s study further proved our results, knockdown of MLLT10 could also inhibit EMT and affect the development of colorectal cancer [24].

In our study, MLLT10 expression could affect the degree of infiltration of immune cells. After regulating the expression of MLLT10, the proportion of CD16 and CD11b positive cells decreased. The abundance of tumor-infiltrating immune cells is highly correlated with the progression of CRC [41]. Our study found that the proportion of CD3 positive cells (T cells) decreased after MLLT10 silencing. It is suggested that MLLT10 could affect the infiltration degree of T cells in CRC. Studies have shown that the proportions of T cells, NK cells, and macrophages in CRC are higher than those in normal tissues [41]. CD3 (-) CD16 (+) are cytotoxic natural killer cells (NK) that can directly kill tumor cells [42]. In the peripheral blood of CRC patients, it was identified that CRC patients with high CD16 (+) NKT-like cells had shorter disease-free survival [43]. That is, relative CD16 (+) NKT-like cells are reduced in patients with high survival. These findings are similar to ours. Low levels of MLLT10 have a low degree of immune cell infiltration.

## 5. Conclusion

Lnc-S100B-2 was screened out in this study, which is closely associated with a poor prognosis of CRC. Regulation of Lnc-S100B-2 and its downstream MLLT10 can affect CRC cell apoptosis. Lnc-S100B-2 and MLLT10 are associated with EMT and immune cell infiltration in CRC cells. It might provide a potential biomarker for CRC prognosis.

## Figures and Tables

**Table 1:** The primers sequences in the study.

Name	Sequences (5‘-3')		
Hsa-miR-331-3p	F	GCCCCTGGGCCTATCCTAGAA	
	RT	GCTGTCAACGATACGCTACGTAAC	
U6	F	CTCGCTTCGGCAGCACA	Product length 94 bp
	R	AACGCTTCACGAATTTGCGT	
Lnc-S100B-2	F	AAGCGACAACCCCTACGAG	Product length 172 bp
	R	CTCCCCACAACAGAAACGTCA	
MLLT10	F	ATGTTCAGGGGAATTTTAAAGTCAA	Product length 100 bp
	R	TGTTACAGAATAACAACCAGTGGG	
Ki67	F	AAGAAGCCCATGAAGACCTCC	Product length 170 bp
	R	CTCTTCTGCCCTCCGCTCT	
Caspase3	F	TGGCAACAGAATTTGAGTCCT	Product length 161 bp
	R	ACCATCTTCTCACTTGGCAT	
Caspase9	F	AAGCCAACCCTAGAAAACCTTACCC	Product length 126 bp
	R	AGCACCGACATCACCAAATCCTC	
Bcl-2	F	AGCTGCACCTGACGCCCTT	Product length 147 bp
	R	ACATCTCCCGGTTGACGCTCT	
PCNA	F	TAGCTCCAGCGGTGTAAACCT	Product length 243 bp
	R	ACTTTCTCCTGGTTTGGTGCTT	
Bax	F	TCACTGAAGCGACTGATGTCCC	Product length 96 bp
	R	ACTCCCGCCACAAAGATGGTC	
N-cadherin	F	TGCCCCTCAAGTGTTACCTC	Product length 182 bp
	R	CAAAATCACCATTAAGCCGAGT	
E-cadherin	F	ATTTTTCCCTCGACACCCGAT	Product length 109 bp
	R	TCCCAGGCGTAGACCAAGA	
Vimentin	F	CCCTTGACATTGAGATTGCCACC	Product length 166 bp
	R	ACCGTCTTAATCAGAAGTGTCCT	
*β*-Catenin	F	ATTCTTGGCTATTACGACAGACT	Product length 176 bp
	R	AGCAGACAGATAGCACCTT	
Snail	F	CGTCCTTCTCCTCTACTTCAGTC	Product length 125 bp
	R	CTTTCGAGCCTGGAGATCCTT	
Slug	F	AGGACACATTAGAACTCACACGG	Product length 196 bp
	R	TACACAGCAGCCAGATTCCTC	
*β*-Actin	F	ACCCTGAAGTACCCCATCGAG	Product length 224 bp
\	R	AGCACAGCCTGGATAGCAAC	

## Data Availability

The data used to support the findings of this study are available from the corresponding author upon request.
